# Supplementary material for: Are carbon emissions trading and green financial instruments synergistic? -Comprehensive quantitative research based on content analysis
Source: PLoS One. 2024 Mar 7;19(3):e0298601. doi: 10.1371/journal.pone.0298601 (PMC10919601; doi:10.1371/journal.pone.0298601)
Supplement: S1 Data — (ZIP) [file pone.0298601.s001.zip › Data/地级市Z_F工作报告.zip!/╜¡╬≈/╥╦┤║╩╨/└┤╫╘╣1⁄2╓┌║┼í╛┬φ┐╦╩2╛▌═°í┐_2006.docx]

各位代表：

现在，我代表市人民政府向大会作政府工作报告，请予审议，并请市政协委员和列席会议的同志提出意见。

一、2005年政府工作回顾

2005年是“十五”时期最后一年，也是推进“和谐创业、富民兴市、加速崛起”的重要一年。我们在省委、省政府和市委的正确领导下，以邓小平理论和“三个代表”重要思想为指导，牢固树立科学发展观，坚持先进性教育与经济社会发展“两手抓、两不误、两促进”，在提前一年完成“十五”计划主要目标基础上，又迈出了经济社会发展的新步伐。

——坚持速度与效益并重，经济发展的自主性和协调性继续改善，呈现出运行平稳、增长较快、效益提升的良好态势

国民经济快速增长。完成生产总值372.2亿元，增长13.2%，三次产业比重由2004年28.8：38.8：32.4调整为26.3：42.0：31.7。克服政策性减收2亿多元的压力，完成财政总收入30.92亿元，增长22.1%，其中地方财政收入18.16亿元，增长17.5%；市本级财政总收入3.08亿元，增长28.4%。固定资产投资保持较快增长，消费需求稳中趋旺，全社会固定资产投资166.7亿元，增长37.3%；社会消费品零售总额114.96亿元，增长13.3%。

工业运行质量稳步提高。规模以上工业完成增加值64.45亿元，增长25.4%，实现销售收入190.5亿元，增长46.8%；利税总额20.49亿元，增长68.9%。规模以上电力能源、建材、机电、食品、医药五大产业，完成增加值43.16亿元，增长28.3%，占全部工业的比重达66.94%。工业园区集聚效应进一步显现，累计入园企业818户、投产企业495户，实现销售收入139.2亿元，增长83.6%；实现税收6.92亿元，增长45.7%。质量兴市、名牌兴企工作取得新进展，3个产品获“国家免检”称号，8个产品获省名牌产品称号，高安市获全省质量兴市先进单位，宜春出入境检验检疫局创立的诚信企业监管模式在全省系统内推广。

农业基础地位进一步巩固。落实粮食直补和良种补贴资金1.54亿元，农机购置补贴资金390余万元。粮食播种面积873万亩，增加7.4万亩，粮食总产326.2万吨，增加9.76万吨，养殖业占农业总产值的比重和高效经济作物占种植业的比重分别达到45.8%和35.9%，分别提高1.5和3.9个百分点。农业产业化主导产业实现综合产值45.58亿元，增长47%；实现税收1.53亿元，增长41.3%，“三品”农业基地面积扩大30%，省级农业产业化龙头企业达19户，万载县被评为全国农业标准化示范区先进单位，奉新县被评为“全国农技推广先进单位”。高产油茶基地建设项目开始启动，以杨树为主的速生丰产林工程大规模展开，栽种杨树701万株，发展速生丰产林工作成为我国南方地区的典范。

——深入推进改革开放，全民创业氛围日渐浓厚，经济发展的内在活力进一步显现

对外开放再创佳绩。制定实施了重大项目招商三年规划，实际引进市外工业大项目资金73亿元，增长108%；实际利用外资2.12亿美元，增长64.2%；出口创汇1.1亿美元，增长85.4%。引进投资超亿元内资工业项目21个，超1000万美元外资工业项目10个。市本级和丰城市两个境外劳务输出基地获准设立。“三争”工作成果丰硕，争取上级各类资金5.99亿元，其中无偿资金4.48亿元。对外交流更加频繁，与韩国尚州市正式缔结友好城市。

民营经济继续壮大。进一步放宽放活有关政策，民营经济得到较快发展。全市私营企业达6849户，增长12.3%；注册资金93.92亿元，增长28.4%。民营企业实现增加值101亿元，增长55.4%；实现销售收入326亿元，增长49.9%；实现税收16.8亿元，增长58.4%，占全市财政总收入的54.4%。其中，纳税100万元以上的民营企业300户，纳税总额达9.72亿元。

各项改革深入推进。江麻公司破产终结，被民营企业收购后恢复生产并实现盈利；长青公司政策性破产准备工作正在抓紧进行。市本级新的国有资产监管运营框架正着手构建。市直事业单位改革分期分批稳步推开，深化乡镇机构改革试点工作已经启动。农村税费改革进一步深化，相关配套改革稳步推进。林业产权制度改革全面展开，配套改革经验在全省推广，整体进度和质量居全省前列。水利工程管理体制改革等正在抓紧实施。

——切实加强基础设施建设，重点项目实施顺利，发展后劲进一步提升

重点项目建设取得突破。在交通、水利等领域确定了27个重点项目，完成投资27.86亿元。宜春机场建设项目列入省“十一五”规划，选址工作已经开始。中心城防洪工程、丰城大联圩、筠安堤等重点防洪工程正抓紧施工，一批重点小（一）型病险水库加固工程陆续开工。全面启动了解决6.3万人的农村饮水安全项目。完成公路改善工程、老区扶贫公路、国债项目工程256.8公里，新建农村水泥（油）路1164公里，新增通水泥路村委会144个，国家重点项目武吉高速公路宜春段正式动工。

城乡建设管理进一步加强。城镇化率提高1.5个百分点，达到32.5%。中心城建设继续推进，袁河水厂顺利竣工，污水处理厂主体工程正加紧建设，芦洲路和钓台路建设、鼓楼片区改造以及第三期小街小巷改造基本完成。中心城区通过实施综合执法、联创联建和市民自治，面貌显著变化，知名度日渐提高，城市管理工作连续三年列全省第一，先后迎接了国家园林城、全国绿化模范城和省文明城的考评检查并成功通过评审。县市城市创建工作稳步推进，奉新、上高、丰城、宜丰正式申报省级园林城市，其中奉新、上高已通过考核验收。全市完成旧村改造试点476个，得到国家建设部充分肯定；建成省级文明村镇示范点15个，高安八景镇、袁州温汤镇、宜丰潭山镇被评为“全国文明乡镇”。

——统筹经济社会发展，大文化产业开始起步，各项社会事业协调推进

大文化产业发展正式启动。策应中心城“江南生态休闲城市”的战略定位，出台了《关于加快全市大文化产业发展的意见》，初步确立了“一带五区六中心”的总体发展框架。明月山温泉风景名胜区被评为国家级风景名胜区。作为大文化产业的重要方面，旅游业发展提速，全年实现综合收入19.19亿元，增长41%，占全市生产总值5.16%，成为我市第三产业的支柱。

科技、教育和人才工作成效明显。争取省级以上科技项目87项，其中国家级项目12项；新增省级工程技术研究中心1个，省级高新技术企业3户，省级民营科技园4个，省级民营科技企业8户。义务教育“一费制”全面实行，“两基”迎省复查年检全部完成，樟树市被授予“江西省巩固提高‘两基’工作先进市”。全市普通高考二本以上上线人数连续八年居全省第一。中心城区部分学校结构调整稳妥推进。高安师范成建制并入宜春学院，宜春学院和宜春职业技术学院在校生规模分别达1.6万人和1.1万人。人才工作有新进展，已选拔出6名有突出贡献的专业技术人才、65名学术学科带头人、315名优秀青年后备人才。

文化、卫生和体育工作成绩斐然。在全省率先启动农村三大文化活动，万载傩舞踩街表演和傩面具展览夺得“中国江西国际傩文化艺术周”金奖。市、县疾病预防控制体系、医疗救治体系基本建成，农村卫生工作和结核病防治工作跃居全省前列，奉新县通过省级卫生城达标验收。广播电视“村村通”工程进展顺利。成功举办了第二届市运会，组织承办了10项全国比赛和5项全省比赛，我市运动员在第十届全运会上夺得5枚金牌。

人口、资源和环境工作进一步加强。人口和计划生育工作全面实现省政府下达的目标，出生人口性别比专项整治取得阶段性成果，农村部分计划生育家庭奖励扶助制度试点经验在全省推广。土地整理项目全面实施，基本农田保护率达85.5%，耕地总量继续保持动态平衡。生态环境建设进一步加强，取缔、关停违法排污企业31户；全市七条主要河流水质明显改善，中心城空气质量由2004年的三级提高到二级。农村新建户用沼气池1.05万个，户用沼气池保有量达3.6万个，并率先在全省成功利用沼气发电。

——突出以人为本，认真解决群众切身利益问题，社会和谐稳定得到有效维护

人民生活水平稳步提高。城镇居民人均可支配收入8230元,增加1098元；农民人均纯收入3430元，增加266元。城乡居民人均储蓄存款5525.5元，增加791元。城乡居民人均消费性支出分别增长25%和20.6%，家庭耐用消费品拥有量以及文化、教育、旅游、保健等消费支出均有不同程度的增长；城乡居民人均住房面积分别达到26.98平方米和35.03平方米，分别增加3.97平方米、0.57平方米，住房质量和配套性能进一步提高。

就业和再就业工作扎实有效。全面开展了“就业政策实效行动”，市、县、街道（乡镇）、社区四级就业信息网络基本建立。全市新增从业人员6.2万人，下岗失业人员再就业3.17万人，免费再就业培训1.5万人，发放再就业小额贷款3309万元，跨省劳务输出70万人。小额贷款信用社区试点建设经验，得到国家劳动和社会保障部充分肯定并向全国推介。

社会保障能力进一步增强。全市参加养老保险21.96万人，征缴基本养老保险费3.86亿元；参加失业保险23.6万人，征缴基金3216万元；参加医疗保险24.8万人，征缴基金1.42亿元。离退休养老金和复员转业人员生活补助得到提高，并按时足额发放。拨付城市低保资金8004万元，保障对象12.2万人。加强了城乡社会救助，下拨各类救助资金4616万余元。投入资金1.1亿元，新建和改建农村敬老院、社会福利院、光荣院19万平方米。“十五”期间实施的434个扶贫开发项目全部竣工并通过验收；争取上级救灾救济款5122.5万元，发放结对帮扶资金484.6万元。

损害群众利益的问题得到有效治理。落实建筑行业民工工资支付保障金制度，清理拖欠农民工工资2123万元，清欠率98.1%。妥善处理群众来信来访，对征地补偿、城镇拆迁、医药采购和医疗服务等方面损害群众利益的突出问题，予以坚决纠正。安全生产保持平稳态势，事故起数和死亡人数大幅下降，实现了“两杜绝一减少”的控制目标。消防安全连续7年未发生重特大火灾事故。加强了社会治安综合治理，依法严厉打击各种刑事犯罪，集中整治突出治安问题，人民群众的安全感进一步增强，社会稳定祥和。

——以打造效能政府为核心，进一步加强民主法制建设，发展环境继续改善

环境创优活动成效明显。深入开展“规范服务效能年”活动，市、县两级经济发展服务中心形成网络，服务功能进一步增强。41个市直单位行政处罚裁量权得到初步规范，对全市行政事业性收费和“小金库”进行了专项清理。加强了领导干部任中经济责任审计和离任审计。组建了市行政投诉中心，制定下发了《进一步优化经济发展环境的若干规定》，对损害发展环境的市直部门实行“一票否决”,经济发展环境得到进一步优化。

民主法制建设进一步加强。深入推进政务公开，自觉接受人大法律监督、政协民主监督和社会舆论监督，人大代表建议、政协委员提案155件全部办复。严格执行重大行政决策和规范性文件法律审查制度，重大决策基本程序更加规范。政府廉政建设和依法行政意识进一步增强。更加重视国防力量建设，民兵预备役建设有新的进步。国家安全、人民防空、民族宗教、统计、编制、人民调解、地震、气象、档案、史志、新闻出版、社会科学、扶残、老龄、红十字会和妇女儿童工作等各项事业都取得了新的成绩。同时，在深入调查研究、充分发扬民主的基础上，认真制定了《宜春市国民经济和社会发展第十一个五年规划纲要（草案）》，为未来五年宜春经济社会发展理清了思路。《纲要（草案）》将提请大会审议。

各位代表，回首过去的一年，成绩来之不易，这是市委正确领导、统揽全局的结果，是市人大、市政协有效监督和支持的结果，是全市人民团结奋斗、奋力拼搏的结果。我谨代表市人民政府，向辛勤工作在各条战线的广大干部群众，向给予政府工作积极支持的人大代表和政协委员致以崇高的敬意！向参与、支持和关心宜春发展的驻宜人民解放军、武警部队、消防官兵、中央和省驻宜单位、各民主党派、工商联、无党派人士、社会团体、离退休老同志和海内外朋友，表示衷心感谢！

在充分肯定成绩的同时，我们也清醒地看到，我市经济社会发展和政府工作还存在不少困难和问题：经济总量小，人均水平低，结构性矛盾较为突出的状况尚未得到根本改观；市本级经济实力偏弱，辐射带动力不强；就业和社会保障压力很大，部分群众生活还比较困难，农民增收难度大；政府机关中服务意识不强、行政效率不高等问题还依然存在，经济发展软环境尚需进一步优化，等等。对这些问题,我们将高度重视，采取更为有力的措施认真加以解决。

二、2006年政府工作的目标要求和工作重点

2006年是实施“十一五”规划的开局之年。做好今年各项工作，对于顺利实施全市“十一五”规划、加快全面建设小康宜春步伐，具有十分重要的意义。根据中央经济工作会议和省委十一届十次全会精神，2006年政府工作总体要求是：以邓小平理论和“三个代表”重要思想为指导，深入贯彻落实党的十六大和十六届五中全会精神，紧紧抓住重要战略机遇期，立足科学发展，推进依法行政，加快改革创新，促进快速崛起，发挥开放型经济推动、市场拉动、大文化产业带动作用，做强工业，做旺人气；坚持分类指导，大力实施“心圈廊”发展战略，全面提升县域经济发展的整体水平；关注民生，维护稳定，促进社会和谐进步，为全面实现“十一五”规划目标奠定坚实基础。主要预期目标为：生产总值增长12%；财政总收入增长15%，其中地方财政收入增长12%；规模以上工业增加值增长25%；全社会固定资产投资增长20%；实际利用境外资金增长25%，引进市外工业资金增长17%，外贸出口增长25%；社会消费品零售总额增长12%；农民人均纯收入增加200元；城镇居民人均可支配收入增长9%；城镇登记失业率控制在4.5%以内，人口自然增长率控制在7.84‰以内，城镇化率达到33.5%。

实现这一总体要求和目标，必须以科学发展观统领各项工作，突出主攻工业这个中心，依靠开放型经济推动、市场拉动和大文化产业带动，力求经济社会发展有新突破。

（一）突出重大项目招商，全面推进开放型经济

紧紧抓住招商引资这一主线，巩固已有成果，拓展开放领域，促进经济结构优化升级。

继续抓好重大项目招商。充分发挥宜春作为“长珠闽”腹地的区位优势，大力推进体制衔接、产业对接、技术交流和市场融合，精心选择引进与我市产业关联度高、带动性强的大项目，促进产业配套发展，逐步形成一批具有地方特色、支撑力强的产业集群。健全招商引资工作协调机制，完善目标考核、业绩奖惩和跟踪服务办法。积极开展境外招商活动，推动利用外资工作再上新台阶。

进一步扩大对外贸易。优化出口产品结构，巩固传统市场，拓展新兴市场，提高优势产品的国际市场份额。积极争取国家支持政策，重点扶持高新技术企业和农业产业化龙头出口企业扩大出口。积极鼓励和帮助具备条件的企业自营出口。发挥市企业出口促进中心在信息沟通、融资担保等方面的作用，壮大出口企业群体。继续推行管理体系认证、产品认证和产地认证等措施，增强对外贸易的抗风险能力。

积极拓宽开放领域。整合优质资源项目，探索参与国际资本运作新途径，先行启动国有林场、城市供水、工业园区和物流平台等项目，全面摸清情况，做好基础工作，搞好项目策划，努力推向国际资本市场。

（二）加速产业集聚，提高工业化水平

按照“抓工业就要抓支柱产业，抓支柱产业就要抓骨干企业，抓骨干企业就要抓主导产品”的思路，全面提升工业经济运行质量和水平。

坚定不移地抓好园区建设。完善工业园区总体规划和功能分区，确定主导产业，培植园区特色。进一步加强基础设施和生活服务设施配套。更加注重单位土地面积投资强度和产出效益，继续清理“圈而不建”、“圈大建小”项目。加大帮扶力度，提高入园企业的开工率、投产率和达产达标率。进一步抓好市经济技术开发区建设，开展委托招商，搞好跟踪服务，降低开发成本。

培育壮大支柱产业。继续抓好医药、建材、食品、机电和电力能源五大支柱产业，对销售收入上亿元企业和5000万元以上的技改扩建项目，实行一厂一策，重点帮扶。着力抓好仁和集团、济民可信等企业建设，促进红狮水泥、兰丰水泥、华厦水泥、恒泰塑业等项目达产达标，加快旺旺食品扩建、青龙高科技改项目建设，推进江西四特、中绿食品等企业稳步发展，壮大江特公司、华伍电器、南特公司等骨干企业实力，加快丰电二期2×66万KW发电机组建设，抓紧做好洪屏电站前期工作，争取早日开工。积极推进精细化工、纺织服装两大后续产业发展，加快宝成鞋业、富达盐化工、蓝恒达盐化工等重大项目建设。鼓励企业增强自主创新能力，支持帮助企业争取省高新技术产业发展基金。加强煤电油运调度，抓好银企合作信贷项目跟踪落实，搞好重大产业项目的综合协调。深入开展“质量兴企”、“名牌兴企”活动，促进企业发展。

加速发展民营经济。认真落实省、市鼓励全民创业的各项措施，为全民创业创造良好条件。动员鼓励在外宜春籍企业家、优秀中小企业主返乡创业。对年纳税前100名和返乡创业、安置就业做出突出贡献的民营企业进行表彰。引导有条件的企业完善法人治理结构，积极靠大联大，搞好自主创新，提升档次和水平。深入开展企业诚信体系建设，建立健全中小企业信用担保市场运作机制，争取更多的信贷资金支持。加快项目库建设，积极争取国债、省技改贴息资金的扶持。

（三）统筹城乡发展，建设社会主义新农村

按照中央提出的“生产发展、生活宽裕、乡风文明、村容整洁、管理民主”的要求，协调推进社会主义新农村建设，努力培植新产业，树立新风貌，创立新机制，塑造新农民，建设新村镇。

进一步提高农业综合生产能力。继续抓好各项惠农政策的落实，严格执行基本农田保护制度，加强土地开发整理，集中资金建设一批旱涝保收、高产稳产的标准农田。切实加强对高致病性禽流感等重大动物疫病的防控工作，进一步完善动植物防疫检疫、农技推广等农业服务体系建设，健全农业科技信息网络，推广农业生产节本增效技术，培育农机作业服务组织，抓好10个水稻生产全程机械化示范村建设。

继续加快农业结构调整。突出发展高效经济作物，巩固发展养殖业，推进生态畜牧小区建设，努力实现“经济作物产值占种植业产值比重和养殖业产值占农业总产值比重均增长5个百分点以上”的目标。大力发展生态农业和高效农业，积极推行绿色大米、绿色蔬菜、椪柑、猕猴桃等农产品标准化生产模式，加快无公害农产品、绿色食品、有机农产品发展。

积极推进农业产业化经营。扶持壮大青龙高科、仁和、元盛、华英、金源、汇银等龙头企业，稳步发展壮大十大农业主导产业。结合实施退耕还林、长防林、日本政府贷款造林等林业工程建设，加快速生丰产林和高产油茶林基地建设，启动毛竹林低改工程。

努力改善农村生产生活条件。加快农村公路建设，加大旧村改造力度，继续开展文明村镇创建活动。加强农村文化建设，推进广播电视“村村通”工程。继续做好饮水安全工作，抓好规模养殖场环保沼气建设，新建户用沼气池1万个。启动2个以电代燃料县试点项目实施和3个农村电气化县建设。加强农田水利基础设施建设，完成黄金、樟树岭、蒙山等中型病险水库除险加固工程，继续抓好潦河、锦北大型灌区续建配套工程、丰城水利血防项目和大中型机电泵站更新改造。抓好扶贫开发，改善贫困地区生产生活条件。实施“万村千乡”试点工程，促进农村消费。

（四）立足提升品位，构建江南生态休闲城市

按照建设“赣西经济中心城市”和“江南生态休闲城市”的总体要求，完善城市功能，优化城市环境，进一步提升中心城的影响力、辐射力和带动力。

完善基础设施配套。在中心城区，启动老年公寓项目和中山中路片区改造，抓好新行政中心建设，完成明月北路、文体路和芦洲路的延伸工程，完善化成洲湿地公园和袁山公园设施配套；加快污水处理厂建设，确保年底前投入运行；做好垃圾中转站、公厕和供水管网改造，推进供气公司改制改气工作；启动淤泥中转站建设，争取秀江东路、沿河东路至赣西大桥城市防洪工程年内完工；扎实推进宜春机场前期工作，配合做好宜安一级公路和武吉高速公路建设，做好320国道大城至万载一级公路建设前期工作，启动105国道大港至玉湖一级公路建设项目，争取完成高安至胡家坊一级公路和干洲至奉新等20条市养公路建设。

提升城乡建设管理水平。巩固中心城创建成果，推进全国双拥模范城创建活动，启动国家卫生城和中国优秀旅游城市创建工作。深入开展联创联建和市民自治，实施精细化管理。加强市容环境和“城中村”的综合整治。深化市政、环卫、绿化综合管护改革。继续开展“环卫精品街”创建活动。严格垄断城市土地一级市场，规范房地产开发，放活交易市场，促进房地产业健康有序发展。加快丰城、樟树、高安中等城市建设步伐，加强对其他县城和省级重点镇、示范镇建设的指导，提高城乡发展融合度，推进新型城镇化。

加快发展大文化产业。抓好中心城区大文化产业发展规划的实施，优先发展旅游、休闲娱乐、体育和传媒业。推进明月山温泉风景名胜区开发建设，修通景区旅游公路，抓好天沐温泉、天工开物园等先导性项目，努力塑造“禅宗圣地”和“富硒温泉”两大品牌。加快百丈山－萝卜潭、洞山、华林寨－上游湖等省级风景名胜区建设，做好明月山温泉风景名胜区和三爪仑国家森林公园申评国家4A级旅游景区工作，争取官山自然保护区进入国家自然保护区行列。推介“中国乡村游”旅游主题，创建农村旅游示范点。

（五）繁荣社会事业，促进经济社会协调发展

在经济增长的基础上，推进各项社会事业，促进社会可持续发展和人的全面进步。

推进科教兴市和人才强市战略。努力构建区域创新体系，加速科技成果转化。强化素质教育，巩固和提高“两基”成果，完善农村义务教育以县为主的管理体制，推进农村寄宿制学校建设和农村现代远程教育工程。大力发展高中阶段教育和幼儿教育。更加注重宜春学院和宜春职业技术学院的建设与发展。积极发展职业教育，支持发展民办教育，重点建设一批服务地方建设的中高等职业学校。大力推进“6666人才工程”，创新人才工作机制，完善人才服务体系，开辟人才引进渠道。

促进公共卫生和体育事业发展。完善市、县两级疾病预防控制体系和公共卫生医疗救治体系，增强突发公共卫生事件应急处理能力，有效控制艾滋病、结核病、血吸虫病等重大传染病。发展城市社区卫生服务，加强农村卫生服务网络建设，稳步推行新型农村合作医疗试点工作。广泛开展全民健身活动，积极争取承办国际国内体育赛事，促进竞技体育和体育产业发展，力争在省第十二届运动会上的成绩继续站前列。

做好资源利用、环境保护和人口计生工作。认真抓好矿产资源开发秩序治理整顿，加强资源节约集约利用。加快生态市建设，编制完成并实施生态市规划。建立和完善各县市环境监控体系，抓好城市污染和工业污染治理，淘汰和取缔严重破坏资源、污染环境和不具备安全生产条件的企业。加强饮用水源保护，继续实施“蓝天、碧水、宁静”三大工程。进一步做好自然灾害的综合减灾工作。严格落实人口和计划生育工作“一票否决”，加强城镇人口和流动人口计划生育管理，规范社会抚养费的征管和使用，加大出生人口性别比整治力度，稳定低生育水平，改善人口结构，提高出生人口素质。

（六）深化各项改革，加快体制机制创新

统筹兼顾，突出重点，加大体制改革和机制创新力度，为加快发展注入新的活力。

扎实推进农村各项改革。进一步巩固农村税费改革成果，确保农民负担不反弹，确保基层组织正常运转，确保农村义务教育经费正常需要。继续推进林业产权制度改革及配套改革，全力抓好林业“三防”体系建设试点和推广工作，加强林地管理，加大木竹加工企业清理整顿力度。推进国有水利工程管理体制改革。继续深化粮食企业改革，建立“国有控股、调控有力、自主经营、效益良好”的粮食经营新机制。

积极推进财政金融和国有资产改革。推进部门预算、国库集中收付和“乡财县代管”改革，实现政府采购管采分离，继续落实预算外资金“收支两条线”规定，收支纳入综合预算或部门预算。严格控制乡村新增债务，坚决杜绝给村级以下下达财政收入任务。健全税源监控体系和征管网络，提高依法治税水平和税收征管质量。优化信贷结构，提高授信额度，积极引进国内外金融机构落户宜春。建立新的国有资产运营监管体系，积极探索资源性资产监管新途径，确保国有资产保值增值。

稳步推进事业单位改革。建立健全以岗位管理为基础的用人机制，力争80%以上的市直事业单位完成人员聘用和其他改革；经营服务类事业单位根据“先易后难、循序渐进、转企改制、一步到位”的原则，年底前基本完成产权改革和身份置换工作。加强对县市区事业单位改革的指导和协调，确保顺利平稳进行。

（七）坚持群众利益至上，更加关注民生

从解决群众最现实、最关心、最直接的问题入手，精心组织实施好四项“民心工程”和六件实事。

1、就业和再就业工程。重点做好“4050”人员再就业工作，确保新增城镇就业4.4万人，下岗失业人员再就业2.1万人，安排财政资金1860万元，购买公益性岗位2800个。加快宜春职业培训公共实训中心建设，培训下岗失业人员4.1万人。

2、社会保障工程。完善城镇职工基本养老保险、基本医疗保险和失业保险制度，扩大工伤、生育保险覆盖面，健全城市居民最低生活保障制度，开展农村低保工作试点。完善城乡特困群体救助制度，妥善解决困难群众子女上学、看病就医等实际问题。

3、社会平安工程。全面落实安全生产责任制，继续深入开展煤矿、花炮等11项安全专项整治，坚决打击非法生产、非法采矿行为。加强社会治安防控体系建设，依法严厉打击各种刑事犯罪和黑恶势力，坚决扫除黄赌毒等社会丑恶现象。做好信访工作，化解基层矛盾，提高处理突发事件的水平。搞好地震、气象、地质灾害预警预报，提高预防自然灾害的能力。

4、食品、药品放心工程。建立健全食品、农产品质量标准体系和检测体系，严格食品、蔬菜、药品等市场准入、质量监督和监测，深化药品流通体制改革。

5、切实维护劳动者合法权益。规范企业用工行为，继续做好清理拖欠工程款和农民工工资工作。

6、继续实施中心城区小街小巷改造。全年投资300万元，安排140条小街小巷改造，全面完成中心城区小街小巷改造工程。

7、加快农村公路建设步伐。力争水泥（油）路竣工里程达1200公里，全市通水泥（油）路的村委会增加282个。

8、努力解决农村饮水安全问题。投资2205万元，解决6.3万农村人口的饮水安全问题。

9、改造农村中小学危房。多方筹措资金，完成全市农村中小学21.48万平方米的危房改造。

10、抓好乡镇敬老院建设。投资4650万元，完成69所乡镇敬老院建设。

（八）加强自身建设，打造诚信和谐的发展环境

按照“为民、务实、清廉”的要求，切实加强政府自身建设，推进政府职能转变和管理创新。

全面推进依法行政。继续清理行政许可项目，规范部门行政处罚自由裁量权，完善行政执法责任制、过错追究制和考核制。自觉接受人大监督，主动加强与人民政协联系，认真听取各民主党派、工商联、无党派人士和人民团体的意见。认真办理人大代表、政协委员的建议和提案。进一步理顺政府职能，强化社会管理和公共服务，建立完善突发公共事件的预警和应急机制。畅通信访渠道，做好矛盾纠纷排查和调解工作，坚决制止和纠正农村征地、拆迁等工作中出现的违规违法行为。

切实加强廉政建设。深入贯彻《公务员法》，严格廉政建设责任制。各级政府和各部门领导要模范遵守党纪国法，带头做到勤政廉政，自觉接受各方面的监督。建立健全教育、制度、监督并重的惩治和预防腐败体系，继续推进领导干部任中经济责任审计工作。把损害群众利益的突出问题作为反腐倡廉工作的重点，坚决查处违纪违法案件，认真纠正损害群众利益的不正之风。进一步搞好政务公开，建设“阳光政府”。加强公务员的学习和教育，提高公务员素质。时刻牢记“两个务必”，坚持勤俭办事业。

不断优化发展环境。全面开展“和谐环境诚信年”活动，加强诚信体系建设，增强政府公信力。进一步加强市、县两级经济发展服务中心建设，切实提高部门服务效能，完善金融、保险、信息、中介评估、物流运输等服务配套，努力构建覆盖各行各业的服务体系。健全经济发展环境考评机制和责任追究制度，坚决从严查处各类损害经济发展环境的人和事。

支持驻宜解放军、武警部队和预备役建设，进一步加强国防后备力量，妥善解决好复员转业退伍军人的安置问题。依法管理宗教事务，抓好《宜春市志》的编修工作。人防、外事、侨务、新闻出版、档案、妇女、儿童、扶残、红十字会等工作都要有新的进步。

各位代表、同志们，站在新的起点，展望宜春的未来，我们深感责任重大，使命光荣。全市人民对政府的信任和支持，给了我们无穷的力量和必胜的信心。让我们紧密团结在以胡锦涛同志为总书记的党中央周围，在市委的坚强领导下，高扬“树正气、讲团结、求发展”主旋律，紧紧依靠全市人民的智慧和力量，聚精会神，和衷共济，圆满完成全年目标任务，在全面建设小康社会的伟大事业中取得新的更大的成绩！
